# Pregnancy Luteoma in Ectopic Pregnancy: A Case Report

**Published:** 2017

**Authors:** Rupinder Kaur Brar, Jyotsna Naresh Bharti, Jitendra Singh Nigam, Sahil Sehgal, Hena Paul Singh, Pushpanjali Ojha

**Affiliations:** 1- Andaman and Nicobar Islands Institute of Medical Sciences, Port Blair, India; 2- Dayan and Medical College and Hospital, Punjab, India

**Keywords:** Ectopic pregnancy, Ovarian masses, Pregnancy luteoma

## Abstract

**Background:**

Pregnancy luteoma is a rare non neoplastic condition of the ovary. It is usually asymptomatic and found incidentally during imaging in pregnancy or during cesarean section. Pregnancy luteoma can also occur after ectopic pregnancy.

**Case Presentation:**

A 30 year old female presented to G.B. Pant Hospital, Andaman and Nicobar Islands institute of Medical Sciences, Port Blair in October 2015 with abdominal pain. After initial investigations, exploratory laporotomy was done for ruptured ectopic pregnancy. Enlarged ovary was removed along with the ruptured portion of fallopian tube. Histopathological examination revealed solid aggregates of large cells with abundant eosinophilic cytoplasm; diagnosis of pregnancy luteoma was given.

**Conclusion:**

It must be considered in the differential diagnosis of ovarian masses in pregnant females that early diagnosis of this entity may avoid unnecessary radical surgery.

## Introduction

Pregnancy luteoma is a rare condition of ovary that is preceded by pregnancy. It is a distinctive, non-neoplastic lesion of ovary, characterized by solid proliferations of luteinized cells, resulting in a tumor-like ovarian enlargement that regresses during the puerperium (1). Most patients are asymptomatic, and the ovarian enlargement is discovered incidently at cesarean section or postpartum tubal ligation. Sometimes, these are associated with mild degree of virilization (2). It can be a diagnostic challenge as it mimics ovarian malignant tumors (3). All of the reported lesions have been benign. Pregnancy luteoma in ectopic pregnancy is rare.

### Case Presentation

A 30 year old female presented to the emergency department of G.B. Pant Hospital, Andaman and Nicobar Islands institute of Medical Sciences, Port Blair in October 2015 with abdominal pain. After taking history, it was found that the patient had amenorrhoea for the last two months. Urine pregnancy test was ordered immediately which came out to be positive. Patient was afebrile but had one episode of vomiting. Complete blood count revealed Hb 10 *gm/dl*, Total leucocyte count 7800/*cumm*, with 67% neutrophils, 28% lymphocytes, 3% monocytes and 2% eosinophils in the differential count, platelet count was 2,30,000/*cumm*. Blood Beta HCG levels were 990 *IU/L*. Ultrasonography was done which revealed ectopic pregnancy. Sonographically, it showed a hypoechoic predominantly solid right ovarian mass containing multiple small cystic areas and a small solid focus. It arose from the right ovary and compressed normal ovarian tissue to the periphery. There was evidence of empty uterus, minimal free fluid in peritoneum and dilated right fallopian tube. The left ovary was sonographically normal.

Patient was shifted to operating theatre and exploratory laporatomy was done for ruptured tubal ectopic pregnancy. Intraoperatively, right ovary was found to be enlarged. It was removed along with the ruptured portion of right fallopian tube. The tissue was sent for histopathological examination in 10% formalin.

### Pathological findings:

**Figure 1.**
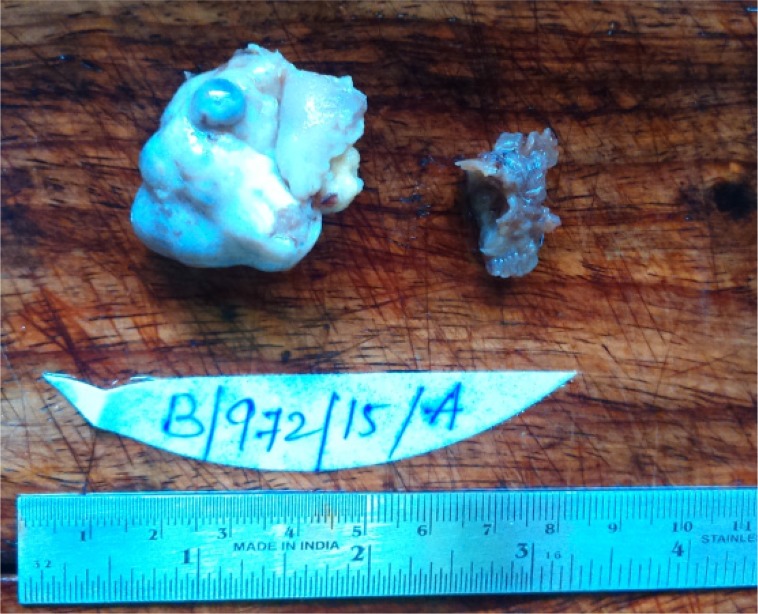
Nodular appearance of enlarged ovary along with a portion of fallopian tube

**Figure 2.**
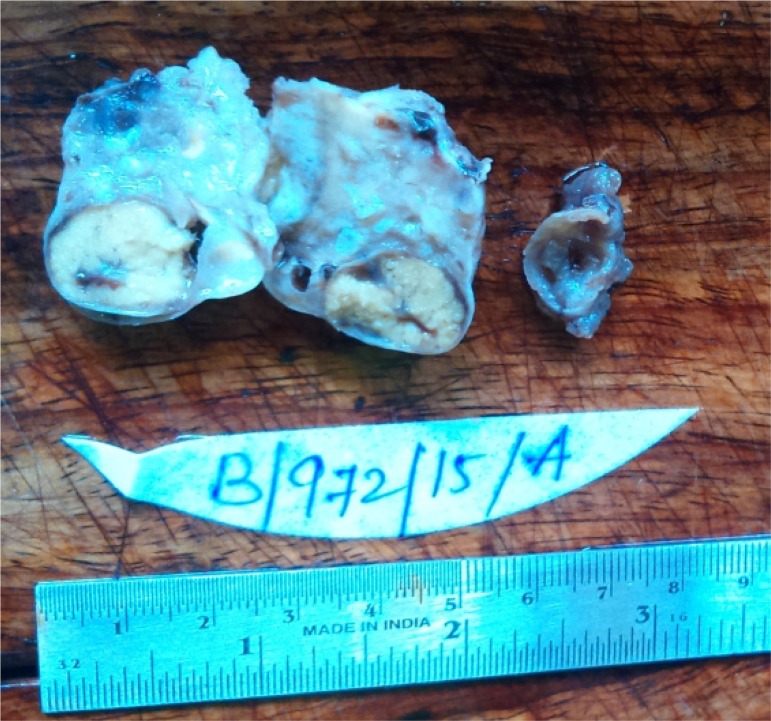
Cut surface showed a large yellow solid nodule

**Figure 3.**
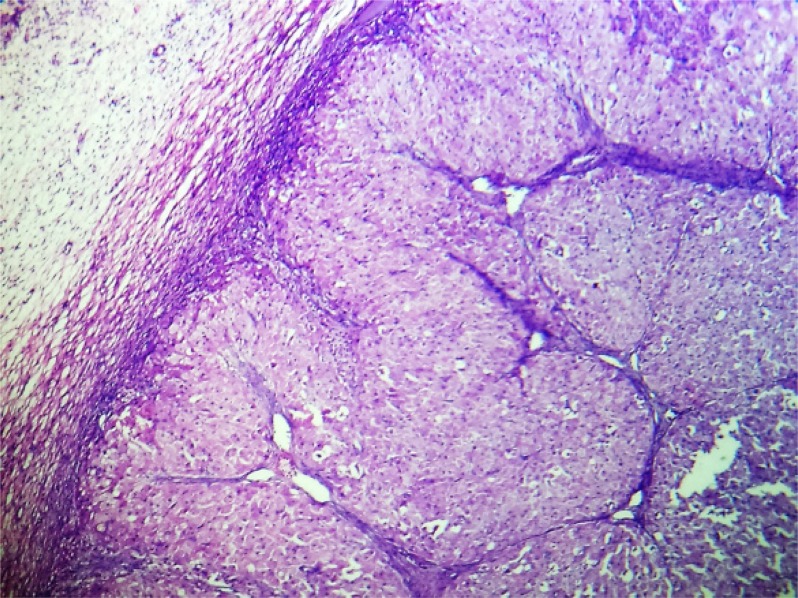
Well circumscribed solid mass of luteinized cells replacing ovarian tissue, seen at the periphery (H&E, ×100)

**Figure 4.**
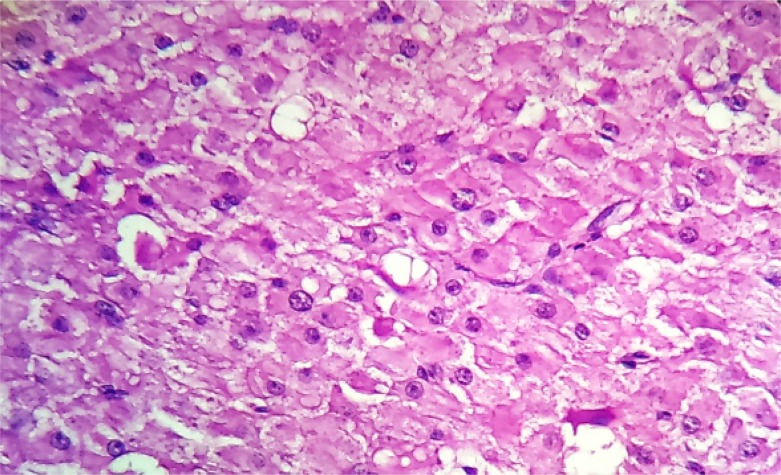
The cells were moderate in size having abundant eosinophilic cytoplasm and central nuclei. Few of these showed prominent nucleoli (H&E, ×400)

Macroscopic examination showed a part of fallopian tube (2 *cm*) filled with clot like material. Also received in the same container was ovary measuring 4.5×4×3 *cm*. Outer surface of ovary was nodular (Figure 1). Cut surface revealed multiple small cysts (0.5–0.6 *cm*) along with a large solid yellow area measuring 2×1.5 *cm* (Figure 2). Microscopic examination after H and E staining revealed inclusion cyst, corpus luteal cyst and focal aggregates of luteinized cells. Sections from the large yellow area showed sharply circumscribed mass of cells arranged in solid growth pattern replacing the normal ovarian parenchyma (Figure 3). The cells were moderate in size with abundant eosinophilic cytoplasm and central nuclei. Few of them showed nucleoli (Figure 4). Features were those of pregnancy luteoma. Sections from fallopian tube showed chorionic villi, haemorrhage, inflammatory cells and decidual tissue, confirming ectopic tubal pregnancy.

## Discussion

Luteoma in ectopic pregnancy is a rare condition and can mimic a solid ovarian neoplasm. It occurs in reproductive age group, can be unilateral or bilateral. Pregnancy luteomas are variable in size, ranging from microscopic upto 20 *cm* in diameter (1). The lesions are multiple in almost half the cases and bilateral in at least one third. Most cases are discovered incidently during cesarean section or post partum ligation of tubes. These are benign, hyperplastic reaction of the theca luetin cells. The difference from theca lutein cyst is that it may sometimes elevate serum androgen levels, presenting the signs and symptoms of virilization. Virilization during pregnancy is rare and it is most commonly caused by pregnancy luteoma or hyperreactio luteinalis (4). Foetus is rarely affected because placenta aromatizes the excess androgens into estrogen. However, there was no virilization in this case.

Incidental pregnancy luteoma has been reported in ovary submitted for ruptured ectopic tubo ovarian pregnancy. It was non encapsulated proliferation of thecal cells in the wall of atretic follicle and had all the pathological findings of late pregnancy luteoma (5). Many cases of pregnancy luteoma have been reported (6). Luteoma of pregnancy must be distinguished from other ovarian masses to avoid oophorectomy in pregnant females (7). Differential diagnosis includes granulosa cell tumor, thecomas, stromal hyperthecosis, unclassified sex cord- stromal tumors, stromal luteomas and hyperreactio luteinalis (8). An association with pregnancy, multiplicity, mitotic activity and absence of cytoplasmic lipids help to distinguish it from steroid cell tumors of ovary.

Resolution of luteoma usually occurs three months postpartum (9). Serum testosterone levels return to normal after 2 weeks postpartum (8). Close clinical monitoring and appropriate follow up is recommended in case of strong clinical suspicion of pregnancy luteoma to avoid any radical surgery (10).

## Conclusion

Pregnancy luteomas depend on hCG stimulation during pregnancy for their structural and functional integrity. These mimic other ovarian masses predominantly the solid and complex cystic masses. Good clinical and imaging features of pregnancy luteoma can obviate the requirement of an unnecessary surgery or termination of pregnancy. Post partum degeneration of nodules has been reported. Pregnancy luteomas can also occur after ectopic pregnancy, though a rare event.
